# Facile engineering of mesoporous silica for the effective removal of anionic dyes from wastewater: Insights from DFT and experimental studies

**DOI:** 10.1016/j.heliyon.2023.e21356

**Published:** 2023-10-20

**Authors:** Ismail Abdulazeez, Ali S. Alrajjal, Saheed Ganiyu, Nadeem Baig, Billel Salhi, Sohaib AbdElazem

**Affiliations:** a Interdisciplinary Research Center for Membranes and Water Security, King Fahd University of Petroleum and Minerals, Dhahran, 31261, Saudi Arabia; b Aerospace Engineering Department, King Fahd University of Petroleum and Minerals, Dhahran, 31261, Saudi Arabia; c Chemistry Department, King Fahd University of Petroleum and Minerals, Dhahran, 31261, Saudi Arabia; d Interdisciplinary Research Center for Refining and Advanced Chemicals, King Fahd University of Petroleum and Minerals, Dhahran, 31261, Saudi Arabia

**Keywords:** Mesoporous silica, SBA-15, Alizarin red, Methyl orange, Methyl red, Bromophenol blue, DFT

## Abstract

The discharge of dye effluents from the textile industries has become a major environmental issue due to its potential to impart serious harm to human health and aquatic life. Mesoporous silica due to its high chemical stability, large surface area, tunable morphologies, large pore volume and pore size and cost-effectiveness is commonly used to remove such dyes before recycling of the wastewater for agricultural, domestic, and industrial applications. However, the low colloidal stability, the fast aggregation of the silica particles and the slow etching of the silica surface often results in the fast deactivation of the adsorbents and limits their long-term applications. In this study, we report the functionalization of mesoporous silica (SBA-15) with ZnO nanoparticles for the effective removal of anionic dyes. The Zn-silica exhibited highly positive surface with a dipole moment of 172 Debye and high charge transfer efficacy with an energy bandgap (ΔE) of 3.35 eV as revealed by quantum chemical DFT simulations. It achieved excellent removal of Alizarin red dye reaching a removal efficiency of 99.99 % and an adsorption capacity of 50 mg/g. In the presence of heavy metal ions commonly present in wastewater (Cd^2+^, Co^2+^, Zn^2+^, Ni^2+^, Cu^2+^ and Hg^2+^), the Zn-silica maintain excellent stability, high selectivity, and reusability within 5 cycles without a significant decline in efficiency. This study thus presents an effective way of wastewater purification on cost-effective adsorbents for meeting the water scarcity demands.

## Highlights

• Porous adsorbent with high surface charge was engineered using combined DFT and experimental studies.
• DFT studies revealed that the adsorbent exhibit high dipole moment and high charge transfer efficacy.
• Excellent removal efficiency of anionic dyes was achieved reaching 99.9 % for alizarin red dye.
• Factors such as pH and contact time during the adsorption were thoroughly optimized.

## Graphical abstract

**Figure:**
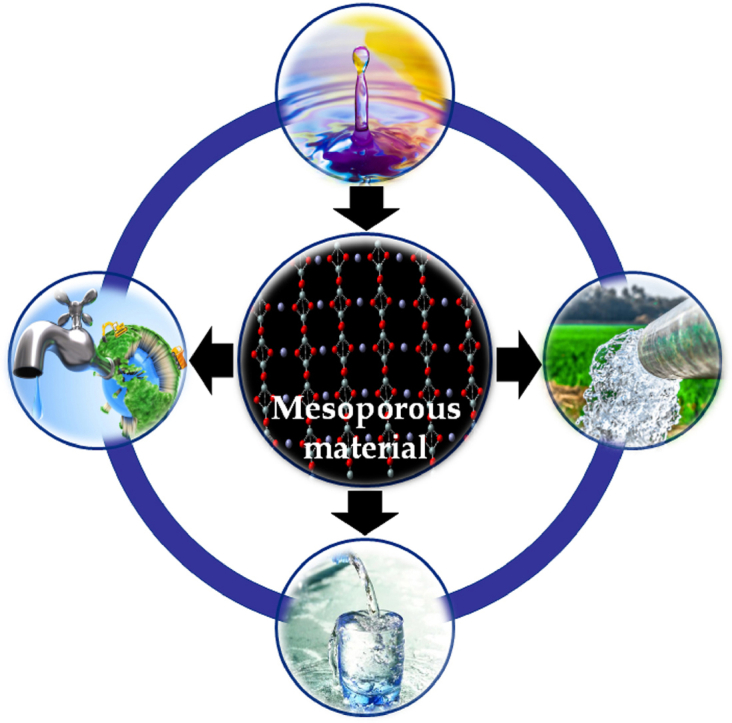

## 1 Introduction

Water is essential for the existence of all living organisms as it serve as a source of food, a habitat for many aquatic species and helps to moderate our climate [1]. While a majority of the Earth's surface amounting to 70 % is covered by water, over 97 % of the water is present in the ocean, while only 1 % is available to humans in the form of groundwater [2]. Extensive industrialization in recent years have contributed significantly to the pollution of water bodies which results in the deterioration of water quality. Waste effluents from pharmaceutical, textiles, cosmetics and plastic industries often contain significant amounts of organic dyes which when released into the environment poses serious threats to terrestrial and aquatic life. These dyes often end up in groundwater and may accumulate in human bodies through the food chain resulting in adverse health-related issues [2]. Their accumulation in water bodies also limits the penetration of sunlight, thereby reducing plants photosynthesis and resulting in lack of plant growth [3]. The treatment of dye-containing wastewater prior to discharge is thus needful for human health protection and ecological sustainability.

Several technologies for water purification such as photo-degradation [4], advanced oxidation [5], membrane separation [6], biological treatment [7] and adsorption [8,9] have been developed. While these technologies have recorded various successes, dye removal via membrane filtration and adsorption which utilizes porous materials are more promising due to the simplicity of operation, high removal efficiency and economic viability [10]. The commonly used porous materials include clay [11], activated carbon [12], silica [13], zeolites [14], polymers [15] and more recently the metal organic frameworks (MOF) [16,17]. Others such as the use of biopolymers, waste biomass, orange peel, neem leaf, cotton wastes, bagasse fly ash, and layered double hydroxides (LDH) have also been reported [[18], [19], [20], [21]]. The low selectivity, low adsorption capacity, slow regeneration kinetics and high cost of synthesis however makes the use of these materials impracticable for industrial wastewater treatment. Thus, efforts are still ongoing in the development of porous wastewater purification materials with tunable properties, excellent removal capacity, high regenerability and a low-cost.

Since their discovery in the early 1990s, mesoporous silica have continued to attract attention due to their unique structural features which include high porosity, high chemical stability, tunable morphologies and cost effectiveness [22]. With the incorporation of templating agents such as the triblock copolymer, P123 into the framework during the synthesis, soon a new class of mesoporous silica which represented a great milestone was discovered. Silica nanomaterials with diverse structural features such as nanosheets [[23], [24], [25]], nanotubes [26,27], rod-shaped [[28], [29], [30]] and hollow spheres [[31], [32], [33], [34]] have since been reported. Numerous studies on the application of these materials in drug delivery [[35], [36], [37], [38]], catalysis [39,40], adsorption and separation [[41], [42], [43], [44]], DNA delivery [45], food science [46] and CO_2_ capture [[47], [48], [49]] have been documented.

Meanwhile, the high surface energy and the inter-particle interactions between the silica units when reacting with pollutants limits their application in dye removal as it often lead to low colloidal stability, fast aggregation and short contact time with the pollutants, which influences the adsorption capacity [50]. Thus, mesoporous silica with various surface functionalities and improved stabilities have been synthesized. The adsorption of cationic and anionic dyes on mesoporous silica functionalized with chitosan [51,52] and amino groups [[53], [54], [55]] have been reported. Due to the strong electrostatic attractions exerted on acidic and basic dyes, mesoporous silica functionalized with amino and carboxylic groups have received greater attentions in recent years due to their rapid adsorption propensities, excellent selectivities and high uptake capacity [[56], [57], [58], [59], [60], [61]]. The incorporation of nanomaterials for enhanced dye adsorption have also been explored [62,63].

Recent reports have shown that the direct contact of dye pollutants with the surface charged groups on silica often results in the slow and irreversible etching of the silica particles [64]. This affects the stability of the pore framework and results in the deactivation of the adsorbents in few cycles of adsorption, which limits their long-term applications. To address this, the incorporation of several metal oxides such as Cu(II) [[65], [66], [67]], Ni(II) [65,68,69], Co(III) [65,69,70], Fe(III) [71], Al(III) [72,73], Mg(II) [69], and Zn(II) [69,74] oxides have shown great improvements in the pore structure and enhanced the removal of various pollutants. While it is generally believed that these improvements were as a result of the electrostatic attractions exerted on the pollutants by these oxides, detailed mechanistic studies supported by quantum chemical DFT simulations on the role of the metal oxides in stabilizing the silica framework and in the removal of the pollutants are still lacking in the literature.

In this study, we report the functionalization of mesoporous silica (SBA-15) with zinc oxide nanoparticles (Zn-SBA) for the effective removal of anionic dyes from wastewater. Quantum chemical density functional theory (DFT) simulations was conducted to reveal the underlying mechanism of the adsorbent-dye interactions leading to the high removal efficiency. The Zn-SBA demonstrated excellent removal of Alizarin red (AR) dye with a removal efficiency of 99.99 % and an uptake of 50 mg/g of adsorbent. The Zn-SBA further maintained excellent stability and selectivity in the presence of common heavy metal ions present in wastewater (Cd^2+^, Co^2+^, Zn^2+^, Ni^2+^, Cu^2+^ and Hg^2+^), and was reusable within 5 cycles with minimal decline in efficiency. The results of DFT simulations revealed that the Zn-SBA exhibited high surface charge with a dipole moment of 172 Debye and high charge transfer efficacy with an energy bandgap (ΔE) of 3.35 eV. It also demonstrated greater attraction for AR with an adsorption energy (E_ads_) of −92.5 kcal/mol, in contrast to methyl orange, methyl red and bromophenol blue with E_ads_ of −55.5, −65.0 and −75.5 kcal/mol, respectively.

## 2 Experimental

### 2.1 Chemicals and reagents

All chemicals and reagents were used as-received without further purification. They include: tetraethyl orthosilicate, TEOS (≥99.0 %), triblock PEG-PPG-PEG co-polymer, P123 (average Mn ∼ 5800), hydrochloric acid, HCl (ACS reagent, 37 %), zinc acetate dihydrate, Zn(CH_3_COO)_2_·2H_2_O (ACS reagent, ≥99.0 %), ethanol (reagent grade, 99.9 %), alizarin red S, AR (ACS reagent, 97 %), methyl orange, MO (ACS reagent, 85 %), methyl red, MR (ACS reagent, 95 %) and bromophenol blue, BPB (ACS reagent, 90 %) all purchased from SIGMA ALDRICH.

### 2.2 Modeling procedure

Structural models of the silica, the zinc-modified silica and the dye molecules were built on GaussView 5.0 graphical user interface and geometrically optimized on Gaussian 09 modeling suite [75] using the density functional theory (DFT) at the hybrid BPW91 functional. The Pople's split valence 6-311G** and the effective core potential LANL2DZ basis sets were chosen for the non-metals and the metal atoms, respectively. A parallel array of interconnected silicon oxide clusters, Si_2_O_2_ [76] was adopted as the silica model, and structurally optimized. Quantum chemical reactivity descriptors derived from the energy of the highest occupied molecular orbital (E_HOMO_) and the lowest un-occupied molecular orbital (E_LUMO_) were calculated following our previous reports [[77], [78], [79]]. All simulations were conducted in aqueous media following the Tomasi's polarized continuum model-self-consistent reaction field (PCM-SCRF) and the solvent was depicted as water [80]. The adsorption of the dye molecules onto the pristine silica and Zn-modified silica were evaluated using equation (1).

1 $$E_{ads}=E_{surface/dye}-\left(E_{surface}+E_{dye}\right)$$

where, E_surface/dye_ represent the free energy of the adsorbent/dye complex, and E_surface_ and E_dye_, the isolated adsorbent surface, and the isolated dye molecules, respectively.

### 2.3 Synthesis of SBA-15

Mesoporous silica (SBA-15) was synthesized following an earlier reported procedure [81] with slight modifications. Briefly, 2 g of P123, 56 ml of 1 M HCl, 4 g of TEOS and 15 g deionized water were thoroughly stirred at 40 °C for 24 h. The mixture was then transferred into a Teflon-lined autoclave, sealed, and heated at 100 °C for 24 h. The resulting solid was collected by centrifuge, washed thoroughly with water and ethanol, and dried at 110 °C overnight. The solid was later calcined in a tubular furnace at 550 °C for 2 h at a heating rate of 2 ^°^C/min.

### 2.4 ZnO modification

The modification was carried out by impregnation with zinc acetate as follows: 0.901 g of zinc acetate was dissolved in 30 ml of ethanol and stirred at 70 °C for 2 h. 0.5 g of SBA-15 was later added with strong stirring and the mixture was dried at 80 °C. The solid was collected and calcined at 550 °C for 2 h at a heating rate of 2 ^°^C/min.

### 2.5 Characterizations

The x-ray diffraction pattern of the SBA-15 and the Zn-SBA were measured on Rigaku Miniflex II x-ray diffractometer using CuKα radiation in the range of 2θ 5–80o. The morphologies were analyzed on Quattro field emission scanning electron microscope (FESEM) fitted with an energy dispersive X-ray spectrometer (Thermo Scientific™). The samples were coated with gold prior to measurements.

### 2.6 Dye adsorption

Batch adsorption of the dye molecules were conducted at room temperature on a digital orbital shaker in 50 ml flasks containing 20 ml of 10 ppm of the dye solutions. 5 mg of the SBA and Zn-SBA were added to the dye solutions with adjusted pH and contact time and the mixture shaken at 200 rpm. The pH of the solutions was adjusted from 3 to 11 using HCl (0.1 M) and NaOH (0.1 M) solutions. All experiments were conducted in triplicate and the average value was reported. At the end of the measurements, the adsorbents were separated from the mixture via centrifuge at 2000 rpm, washed thoroughly in 0.1 M HCl and re-used. The absorbance of the supernatant solution was estimated spectrophotometrically using a pre-established calibration curve (Fig. S1) to determine the residual dye concentrations. The removal efficiency and dye uptake capacity were thus estimated using equations 2, 3), respectively:

2 $$\eta \;\left(\%\right)=\frac{C_{o}-C_{e}}{C_{o}}\times 100$$

3 $$Q_{e}\;\left(mg/g\right)=\frac{\left(C_{o}-C_{e}\right)V}{m}$$

where, C_o_ and C_e_ represent the starting and the equilibrium concentrations of the dye molecules, V is the volume (in L) of the dye solutions and m is the mass (in g) of the adsorbent.

## 3 Results and discussion

### 3.1 DFT modeling

**Fig. 1:**
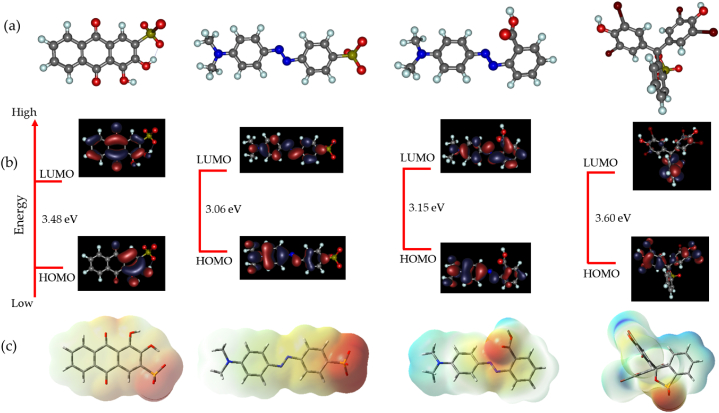
(a) The optimized structural geometries, (b) the frontier orbital distribution and (c) the molecular electrostatic potential maps of (from left to right) AR, MO, MR and BPB dye molecules, calculated at the BPW91/6-311G** level of theory.

**Fig. 2:**
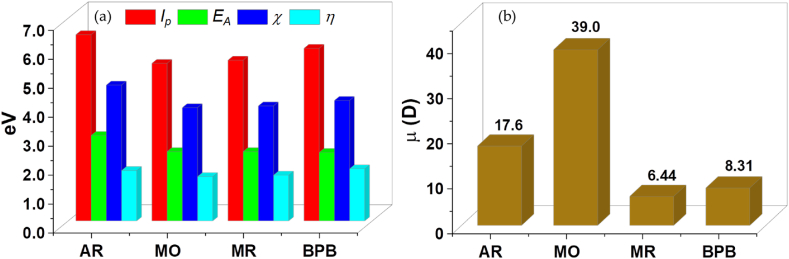
(a) Quantum chemical electronic properties and (b) the dipole moment of AR, MO, MR and BPB dye molecules, calculated at the BPW91/6-311G** level of theory.

The optimized structural geometries, frontier orbital distribution and the molecular electrostatic potential (MEP) maps of the studied dye molecules are presented in Fig. 1a–c. The highest occupied molecular orbitals (HOMO) and the lowest un-occupied molecular orbitals (LUMO) of the dye molecules are fairly distributed along the pi-network and the heteroatoms within the molecules. This implies the tendency of the dye molecules to undergo charge transfer processes involving charge donation or purely electrostatic attractions. Meanwhile, the MEP maps of the molecules indicate that the highest charge donation centers are located on the sulfonate groups of AR, MO, and BPB, while for the MR dye it is centered around the carboxylate group. The HOMO-LUMO energy gap (ΔE) further indicates that MO having a value of 3.06 eV has the potential to undergo donor-acceptor charge transfer processes, in contrast to AR, MR and BPB with ΔE values of 3.48, 3.15 and 3.60 eV, respectively. These results show the charge polarity of the dye molecules and indicates their propensity for studying the adsorption of anionic dyes on the silica and the Zn-silica surfaces. Other electronic properties of the dye molecules such as the global hardness (*η*), electronegativity (*χ*), ionization potential (*I*_p_), electron affinity (*E*_A_) and dipole moment (*μ*) are presented in Fig. 2a and b, and summarized in Table S1. These results suggests that the MO dye having the lowest global hardness of 1.53 eV, the lowest electronegativity of 3.92 eV and the highest dipole moment of 39.0 Debye has the highest potential to donate charges during interactions with the adsorbents.

**Fig. 3:**
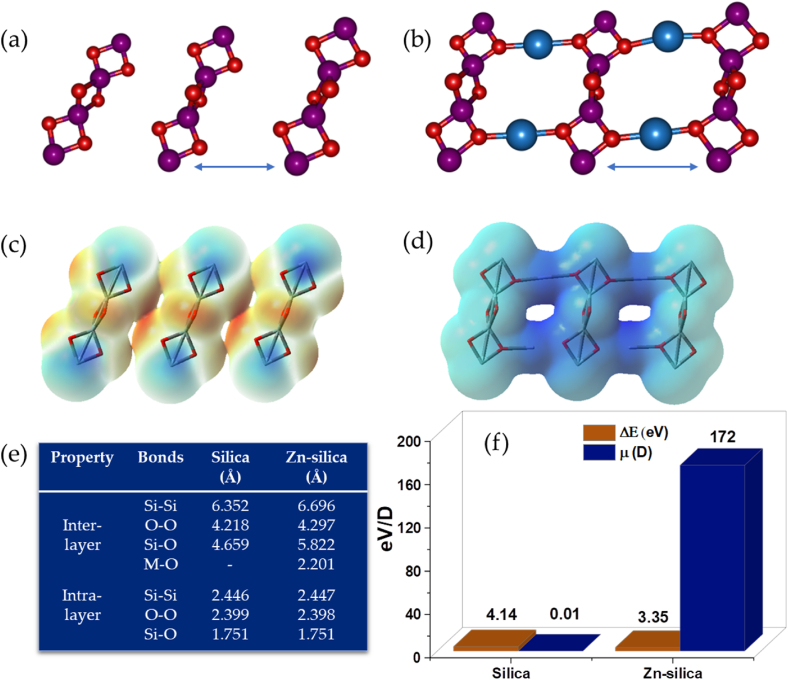
Optimized geometries of (a) silica, (b) Zn-silica, the MEP maps of (c) silica, (d) Zn-silica, the bond properties (e) and the HOMO-LUMO energy gap, and (f) dipole moment of the adsorbent surfaces, calculated at the BPW91/6-311G** and LANL2DZ levels of theory.

Meanwhile, similar structural calculations were conducted on the silica and the Zn-silica surfaces and the results (Fig. 3a–f) revealed that Zn impregnation tunes the electronic properties of the silica by altering the inter and the intra-layer bond properties, increasing the pore openings and significantly increasing the surface charge. The MEP maps indicate high electron density on the pristine silica and the depletion of such on the Zn-silica, making the latter material more susceptible to electrostatic surface attractions when interacting with the dye molecules. The narrowing of the HOMO-LUMO energy gap and the upsurge in the dipole moment upon Zn impregnation further affirms the increase in the dye adsorption potential of the modified surface.

**Fig. 4:**
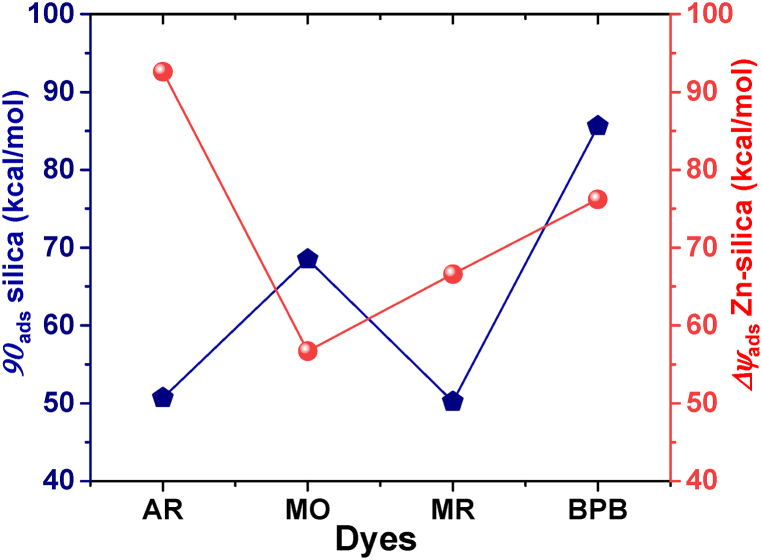
Theoretical adsorption energies (in kcal/mol) of the dye molecules on the silica and the Zn-silica surfaces.

Furthermore, the molecular level interactions of the dye molecules on the surfaces of the pristine silica and the Zn-silica were conducted. The optimized geometries and the frontier orbital distributions of the interactions are presented in Fig. S2 and Fig. S3, while the corresponding adsorption energies (*E*_a_) are presented in Fig. 4. The results indicate that all the dye molecules exhibit high adsorption energies on both the pristine and the modified surfaces with *E*a values of −50.7, −66.5, −50.0 and −85.2 kcal/mol for the AR, MO, MR and BPB dyes, respectively on the silica surface, and −92.5, −55.5, −65.0 and −75.5 for the AR, MO, MR and BPB dyes, respectively on the Zn-silica surface. These results suggest the role of steric effect on the accessibility of the dye molecules to the adsorbent surface as despite MO having high charge donation capacity, it exhibits minimal attraction to the modified surface. It also revealed that the geometry of the AR dye favors the high adsorption potential on the modified surface due to minimal steric hindrance resulting in high surface accessibility. Similarly, due to the high electron density on the carboxylic groups on the MR dye molecules, the strong electrostatic attractions exerted by the Zn-silica surface was able to overcome the steric repulsions, resulting in higher *E*_ads_ compared to the pristine silica surface. The BPB in contrast, exhibited a slight decline in *E*_ads_ due to similar reasons as the MO.

**Fig. 5:**
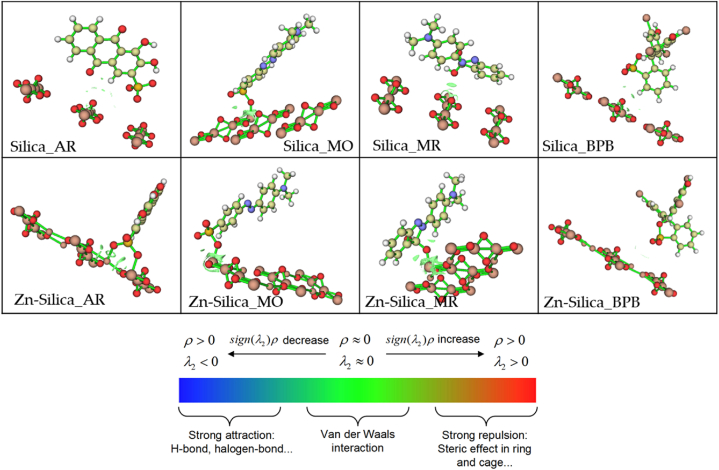
The reduced density gradient (RDG) plots of the interactions of the dye molecules on the silica and the Zn-silica surfaces.

Meanwhile, an inspection of the non-covalent interactions (NCI) of the dye molecules on the surface of the adsorbents revealed the presence of strong van der Waals electrostatic attractive forces with the Zn-silica surface (Fig. 5), in accordance with the Bader's theory of atoms in molecules (AIM) [82]. According to this theory, the nature of interactions between two molecules can be characterized based on the electron density (ρ) values as either van der Waals interaction, strong steric effects, or hydrogen bonds interactions and plotted as the reduced density gradient (RDG) isosurface.

**Fig. 6:**
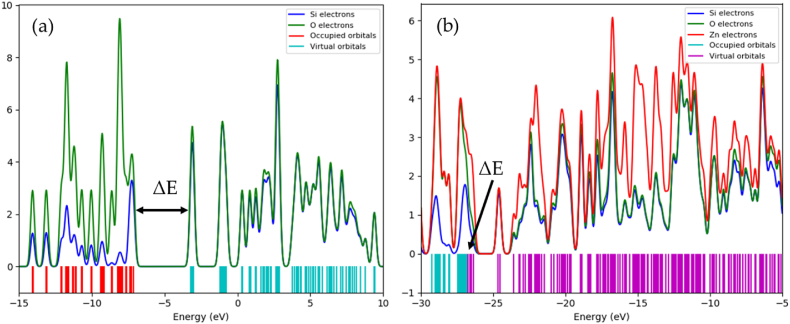
The partial density of states (PDOS) plots of the isolated (a) silica, and (b) Zn-silica surfaces.

Lastly, the electron distribution on the surface of the isolated adsorbents and upon interactions with the dye molecules was investigated by the partial density of states (PDOS) analysis. The PDOS plot of the isolated silica molecule (Fig. 6a) revealed that the HOMO is made largely of the O1s orbital state, whereas the LUMO comprises of the hybridization of the O1s and the Si2p orbital states, with the HOMO-LUMO energy gap (ΔE) of 4.14 eV. A significant alteration is however observed on the Zn-silica surface with the lowering of the peak intensities, the shift of the peaks to more negative values and the lowering of the ΔE to 3.35 eV, all attributed to the non-covalent hybridizations and the overlap of the Zn2p orbitals (Fig. 6b). These changes in the PDOS facilitates the charge mobility on the Zn-silica surface, making it more attractive to the anionic dyes, as reflected in the PDOS plots upon interactions with the dye molecules (Fig. S4 and Fig. S5). These results thus demonstrate the enhancement in the surface charge of silica upon Zn-impregnation, which consequently led to strong electrostatic interactions with the dye molecules.

### 3.2 Synthesis and characterization of adsorbents

**Fig. 7:**
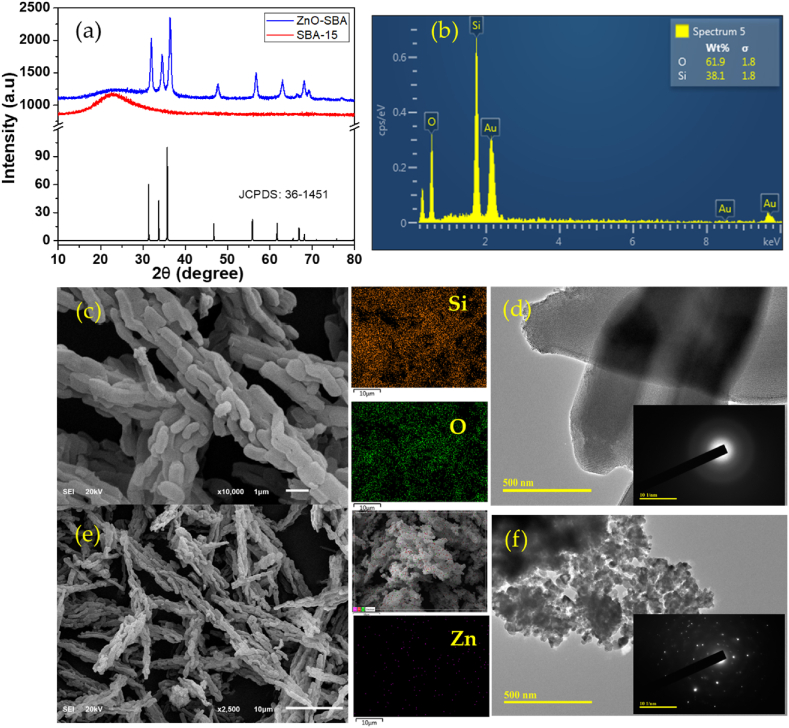
(a) x-ray diffraction pattern of the SBA-15, Zn-SBA and the simulated ZnO, (b) the EDX elemental distribution of the SBA-15, (c) the morphology of the SBA-15 and the elemental mapping showing the Si and O distributions, (d) the TEM image of the SBA-15 with the selected area electron diffractogram (SAED) as inset, (e) the morphology of the Zn-SBA with the distribution of Zn, (f) the TEM and SAED of Zn-SBA.

Mesoporous silica having cylindrical shapes was synthesized and characterized as presented in Fig. 7a–f. Similarly, the Zn modification was successfully carried out as presented. All the x-ray diffraction peaks of the ZnO were detected on the modified Zn-SBA confirming the successful impregnation of SBA-15 with ZnO nanoparticles. The elemental mapping also affirms the modification. The selected area electron diffraction (SAED) images further reveal the amorphous nature of the pristine SBA and the presence of crystalline ZnO nanoparticles on the Zn-SBA, in agreement with the XRD pattern.

### 3.3 Dye removal studies

**Fig. 8:**
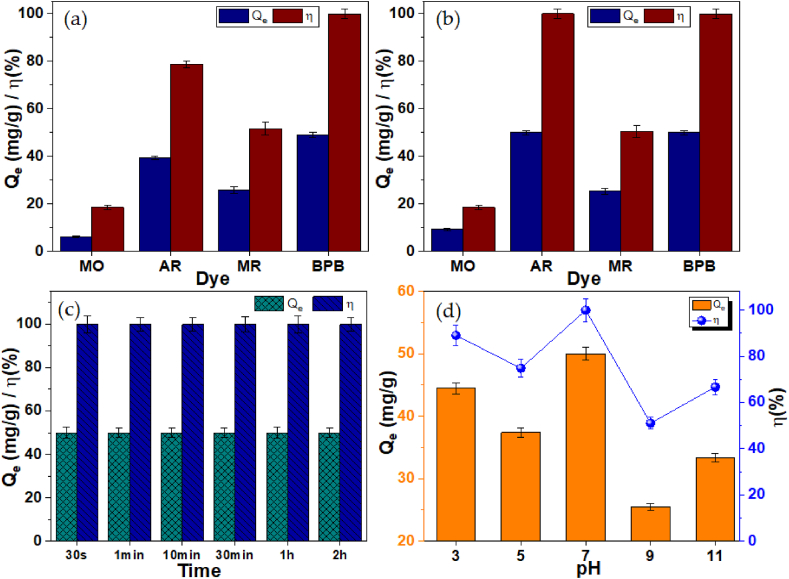
The dye removal efficiencies and adsorption capacity on (a) SBA-15, and (b) Zn-SBA surfaces. The time and pH optimizations on the Zn-SBA are presented in (c) and (d), respectively. (Adsorbent dosage = 5.0 mg, T = 25 ± 1 °C, initial contact time = 1 h, dye concentration = 10 ppm). The error bars represent the standard deviation of the average adsorption capacity/removal efficiency.

The removal of AR, MO, MR and BPB dye molecules on the pristine SBA-15 and the Zn-SBA materials was successfully carried out and presented in Fig. 8a–d. Preliminary studies showed that both the SBA and the Zn-SBA adsorbents exhibits attractions for the dye molecules. However, the AR dye having the minimal steric blockage of accessible adsorption sites on the adsorbent surface has the highest removal efficiency of 99.99 % on the Zn-SBA and was chosen for further studies. Operational parameters such as the dye contact time and the pH of the medium were optimized and the results indicates that the Zn-SBA achieved maximum removal efficiency of 99.99 % in just 30 s, and a maximum uptake of 50 mg/g at the pH 7, making it suitable for practical applications.

**Fig. 9:**
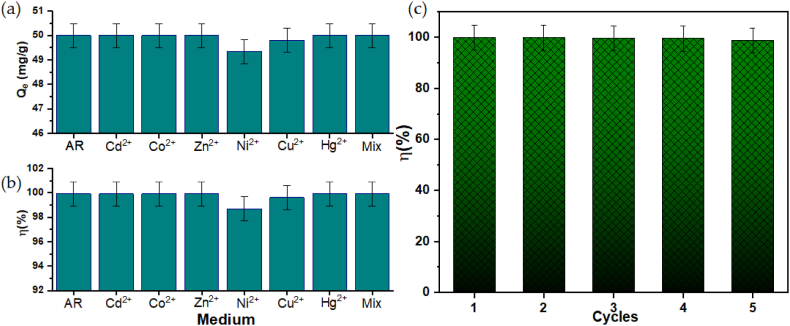
(a) The adsorption capacity, and (b) the removal efficiency of AR dye molecules in the presence of common heavy metal ions present in wastewater. The stability of the removal in 5 cycles is presented in (c). (Adsorbent dosage = 5.0 mg, T = 25 ± 1 °C, contact time = 10 min, dye concentration = 10 ppm, pH = 5). The error bars represent the standard deviation of the average removal efficiency.

The interference of common heavy metal ions present in wastewater on the removal efficiency of AR by Zn-SBA was further explored and the results (Fig. 9a–c) indicate that except for Ni^2+^ and Cu^2+^ ions which exert slight impact on the removal efficiency of AR, the Zn-SBA steadily remove the AR dye molecules in the presence of 10 ppm of the heavy metal ions and maintains a steady uptake. The Zn-SBA further maintained excellent stability in the removal of AR dye molecules within 5 cycles of measurements without a significant decline in efficiency, making it a cost-effective adsorbent for practical wastewater treatment. It is also worthy to mention that the interference posed by the Cu^2+^ ions is insignificant as the decline in adsorption capacity and removal efficiency falls below the 5 % significance level, in contrast to that posed by Ni^2+^ ions which is greater in magnitude. Thus, Ni^2+^ is likely to impact negatively on the removal efficiency of Zn-SBA towards AR dye molecules, probably due to matching orbitals during adsorption, matching electronic properties such as global hardness, and favorable conditions during adsorption such as the pH.

## 4 Conclusion

In conclusion, the removal of anionic dye pollutants in wastewater using zinc impregnated mesoporous silica (Zn-SBA) was successfully explored using first principle DFT simulations and batch adsorption studies. The Zn modification resulted in a highly positive surface having a dipole moment of 172 Debye and the lowering of the bandgap to 3.35 eV. The modified surface exhibited excellent removal of 10 ppm of Alizarin red dye achieving a removal efficiency of 99.99 % in just 30 s contact time, and an uptake capacity of 50 mg/g. The Zn-SBA further maintained excellent removal of AR in the presence of common heavy metal ions present in wastewater and was stable within 5 cycles of reuse. This study presents a cost-effective and efficient way of wastewater purification and could be further explored in practical applications.

## Data availability

The raw/processed data required to reproduce these findings will be made available by the corresponding author upon request.

## CRediT authorship contribution statement

**Ismail Abdulazeez:** Conceptualization, Funding acquisition, Investigation, Methodology, Software, Writing – original draft. **Ali S. Alrajjal:** Methodology, Writing – review & editing. **Saheed Ganiyu:** Conceptualization, Visualization, Writing – review & editing. **Nadeem Baig:** Conceptualization, Data curation, Writing – review & editing. **Billel Salhi:** Resources, Supervision, Writing – review & editing. **Sohaib AbdElazem:** Validation, Writing – review & editing.

## Declaration of competing interest

The authors declare that they have no known competing financial interests or personal relationships that could have appeared to influence the work reported in this paper.
