# Supplementary material for: Facile engineering of mesoporous silica for the effective removal of anionic dyes from wastewater: Insights from DFT and experimental studies
Source: Heliyon. 2023 Oct 20;9(11):e21356. doi: 10.1016/j.heliyon.2023.e21356 (PMC10618791; doi:10.1016/j.heliyon.2023.e21356)
Supplement: Multimedia component 1 [file mmc1.docx]

**Facile engineering of mesoporous silica for the effective removal of anionic dyes from wastewater: insights from DFT and experimental studies**

Ismail Abdulazeez^a^*, Ali S. Alrajjal^b^, Saheed Ganiyu^c,d^, Nadeem Baig^a^, Billel Salhi^a^, Sohaib AbdElazem^a^

*^a^ Interdisciplinary Research Center for Membranes and Water Security, King Fahd University of Petroleum and Minerals, Dhahran 31261, Saudi Arabia.*

*^b^ Aerospace Engineering Department, King Fahd University of Petroleum and Minerals, Dhahran 31261, Saudi Arabia.*

*^c^ Chemistry Department, King Fahd University of Petroleum and Minerals, Dhahran 31261, Saudi Arabia.*

*^d^ Interdisciplinary Research Center for Refining and Advanced Chemicals, King Fahd University of Petroleum and Minerals, Dhahran 31261, Saudi Arabia.*

* Corresponding author: Dr. Ismail Abdulazeez ([ismail.abdulazeez@kfupm.edu.sa](mailto:ismail.abdulazeez@kfupm.edu.sa))

**Supplementary data**

**
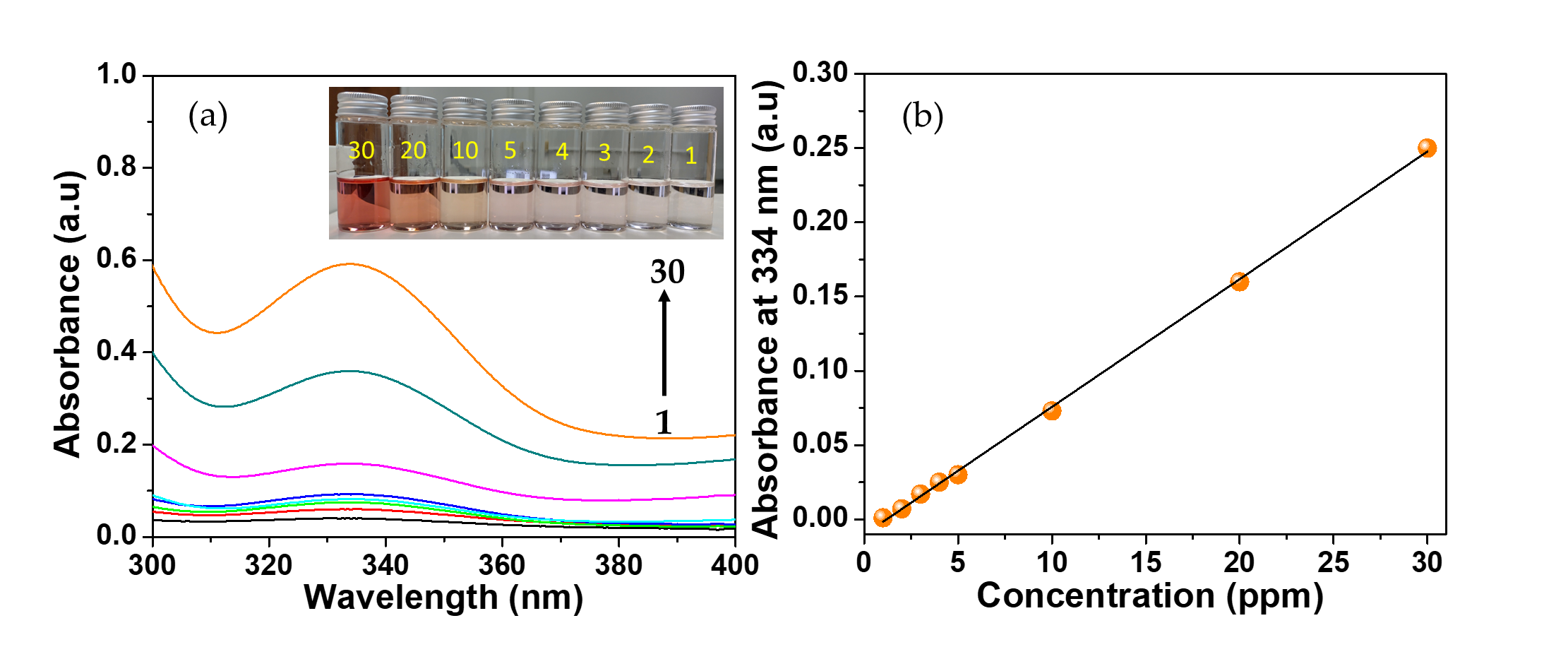
**

**Figure S1.** (a) UV-Vis spectrophotometric response of various concentrations of alizarin red, and (b) the corresponding calibration curve using absorbance at *λ*_max_ 334 nm.

**
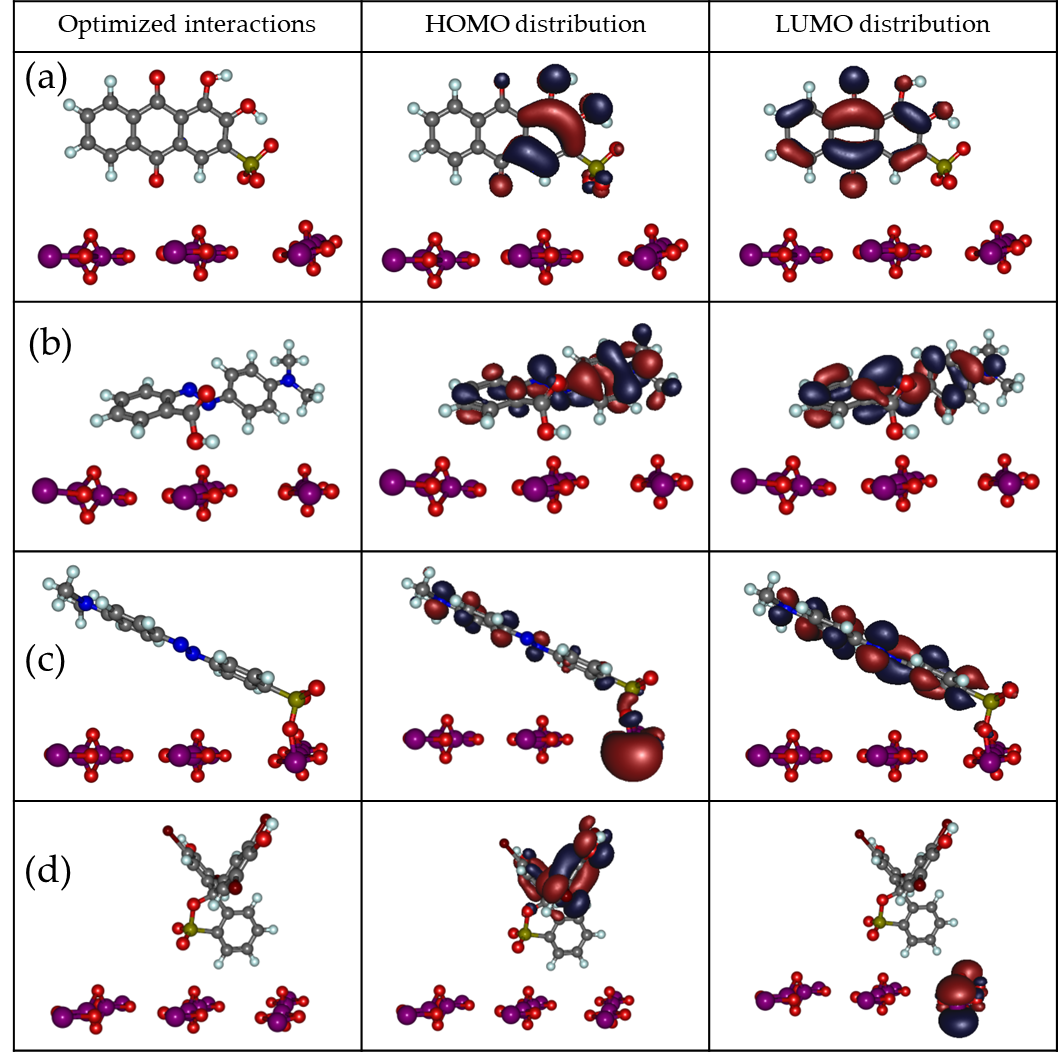
**

**Figure S2.** Molecular level interactions of (a) AR, (b) MR, (c) MO and (d) BPB dye molecules on the surface of pristine silica at the BPW91/6-311G** level of theory.


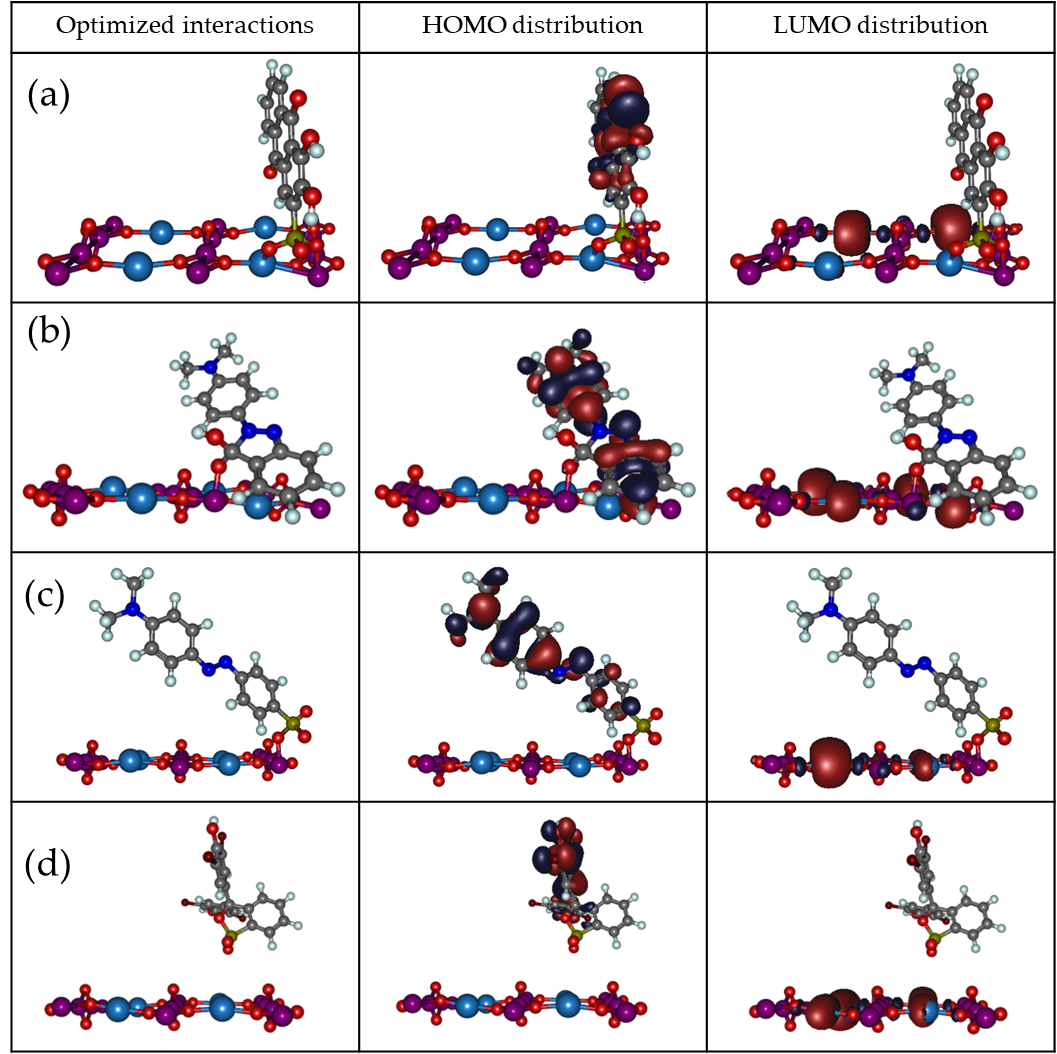


**Figure S3.** Molecular level interactions of (a) AR, (b) MR, (c) MO and (d) BPB dye molecules on the surface of Zn-silica at the BPW91/6-311G** and LANL2DZ level of theory.


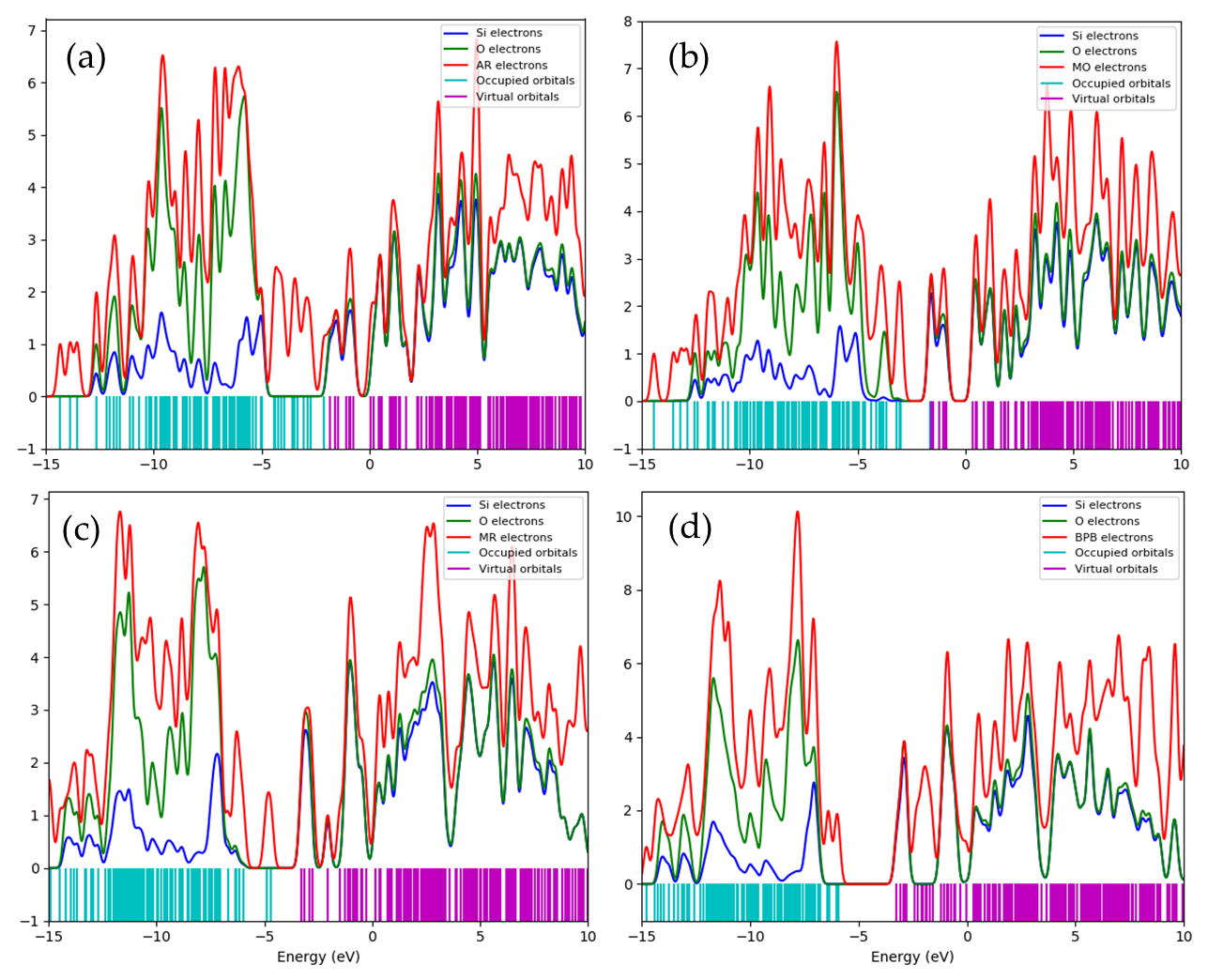


**Figure S4.** The partial density of states (PDOS) plots of the interactions of silica with (a) AR, (b) MO, (c) MR and (d) BPB dye molecules.


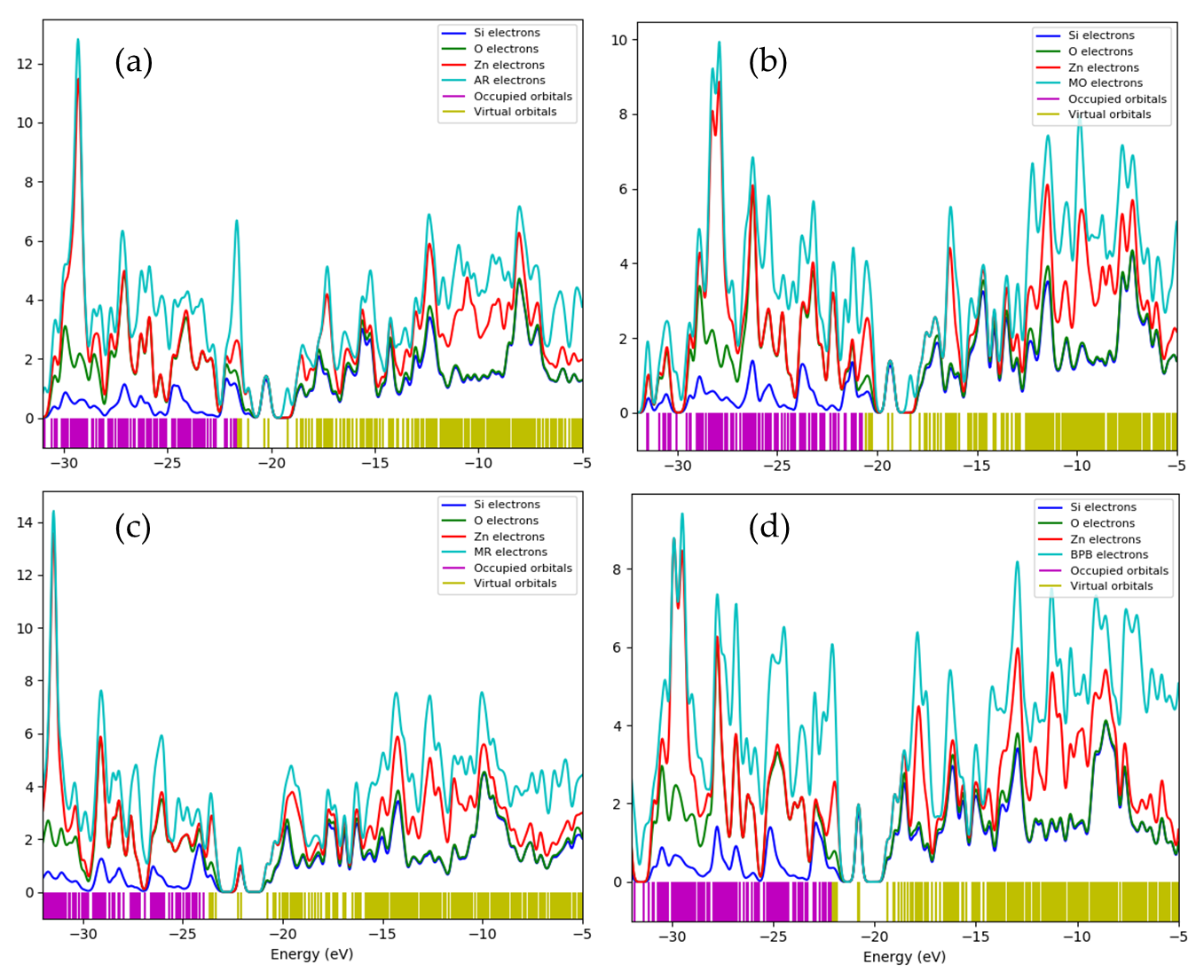


**Figure S5.** The partial density of states (PDOS) plots of the interactions of Zn-silica with (a) AR, (b) MO, (c) MR and (d) BPB dye molecules.

**Table S1**. Electronic properties of the dye molecules calculated at the BPW91/6-311G** level of theory

| Electronic properties (eV) | Alizarin red | Methyl orange | Methyl red | Bromophenol blue |
| --- | --- | --- | --- | --- |
| Ionization potential, *I*_p_  Electron affinity, *E*_A_  Electronegativity, *χ*  Global hardness, *η* | 6.43  2.95  4.69  1.74 | 5.44  2.39  3.91  1.53 | 5.55  2.39  3.97  1.58 | 5.96  2.36  4.16  1.80 |
